# TREM2 in MASH: integrating lipid metabolism and immune response

**DOI:** 10.3389/fimmu.2025.1604837

**Published:** 2025-06-25

**Authors:** Shuyi Shi, Yangyang Zhou, Haiming Zhang, Junli Zhang

**Affiliations:** ^1^ Department of Gastroenterology, Union Hospital, Tongji Medical College, Huazhong University of Science and Technology, Wuhan, China; ^2^ Department of Integrated Traditional Chinese and Western Medicine, The Central Hospital of Wuhan, Tongji Medical College, Huazhong University of Science and Technology, Wuhan, China

**Keywords:** metabolic dysfunction -associated steatohepatitis (MASH), triggering receptor expressed on myeloid cells-2 (TREM2), lipid-associated macrophages (LAMs), lipid metabolism, immune response

## Abstract

Metabolic dysfunction-Associated Steatohepatitis (MASH), a progressive liver disease characterized by disturbances of lipid metabolism and chronic inflammation response in liver has become the most prevalent chronic liver diseases worldwide. Recent single-cell transcriptomic studies in both humans and mice have identified a distinct population of liver macrophages characterized by the expression of Triggering Receptor Expressed on Myeloid Cells 2 (TREM2), also be known as lipid-associated macrophages (LAMs), is highly expressed in macrophages under metabolic stress conditions. Several studies have demonstrated that TREM2+ macrophages play a crucial role in lipid metabolism and immune responses, contributing to the reversal of MASH. This review provides a comprehensive overview of the current evidence on the roles of TREM2+macrophages in regulating lipid metabolism and immune responses in MASH, with emphasis on the functions of TREM2+ macrophages in lipid handling and inflammation regulation, which could provide novel insights into the pathogenesis of MASH and inform targeted therapeutic strategies.

## 1 Introduction

Metabolic dysfunction-Associated Steatohepatitis (MASH) characterized by disturbances of lipid metabolism and chronic inflammation response in liver is a progressive form of Metabolic dysfunction-Associated Steatotic Liver Disease (MASLD), which can further progress to severe hepatic outcomes, including cirrhosis, liver failure, and hepatocellular carcinoma (HCC) (1–4). Over the past decade, it has become evident that the clinical burden of MASH extends beyond liver-related mortality. MASH is now recognized as a multisystem disease that affects several extrahepatic organs, increasing the risk of type 2 diabetes mellitus (T2DM), cardiovascular disease (CVD), cardiac disorders, and chronic kidney disease (CKD) (5).

The “two-hit” hypothesis, which has long been proposed to explain MASH pathogenesis, suggests that, in the context of hepatic steatosis, a second “hit” from factors such as oxidative stress is necessary for the progression to MASH. However, this theory is now considered not comprehensive (6, 7). Current understanding emphasizes that disturbances in energy metabolism and chronic inflammation are two major hallmarks of MASH (8). When the liver’s capacity to process primary metabolic substrates, such as carbohydrates and fatty acids, is overwhelmed, toxic lipid species accumulate (6, 9). These metabolites induce hepatocellular stress, injury, and death, leading to persistent chronic inflammation, fibrogenesis and genomic instability (10–13). It is widely accepted that insulin resistance is a key risk factor for disorders in hepatic fatty acid metabolism (14). Impaired insulin signaling in adipose tissue contributes to MASH by disrupting lipolysis, resulting in the excessive delivery of fatty acids to the liver (6, 15, 16). Disruption of hepatic immune homeostasis is a core mechanism in the pathogenesis of MASH. The liver is not only the center of metabolism but also an important regulator of immune responses. Hepatic immune homeostasis relies on the balance and function of immune system cells, particularly the coordination of macrophages, T cells and hepatic stellate cells (HSCs). The liver has a strong immune tolerance, capable of suppressing excessive immune reactions. However, in MASH, this immune tolerance mechanism is impaired, leading to a persistent inflammatory state in the liver. Activation of the hepatic inflammasome triggers the expression of pro-inflammatory cytokines, such as Interleukin-1β (IL-1β), Interleukin-6 (IL-6), and Tumor Necrosis Factor-α (TNF-α), which promote pyroptosis via caspase-1 activation (17, 18). Furthermore, recent studies in murine models have highlighted that hepatocyte inflammation may represent a critical link between initial metabolic stress, subsequent hepatocyte death, and the stimulation of fibrogenesis in MASH (6, 19).

Liver macrophages, including resident Kupffer cells (KCs) and monocyte-derived macrophages (MoMFs), play pivotal roles in the pathogenesis of hepatic inflammation and fibrogenesis in MASH (20–23). During the progression of MASH, resident KCs phagocytose lipid droplets and dead cell debris, undergoing gradual apoptosis and circulating bone marrow-derived monocytes acquire features of KCs, ultimately assuming their functions (24–28). Due to the important roles in phagocytosis and immune response, targeting macrophages (KCs or MoMFs) has emerged as a central therapeutic strategy for treating and potentially reversing the progression of MASH.

Triggering Receptor Expressed on Myeloid Cells 2 (TREM2), a lipid-binding receptor expressed on the cell surface, has previously been shown to maintain the metabolic fitness of microglia in Alzheimer’s disease (AD) (29–33). Since Xiong et al. conducted transcriptomic analyses in the liver during MASH and identified a population of TREM2-expressing macrophages, which primarily originate from MoMFs, studies have increasingly focused on the role of TREM2 in MASH, particularly in the regulation of metabolism, immune response and fibrosis, with the aim of identifying new and effective drug targets for MASH (34). Moreover, latest research conducted by De Ponti et al. have also indicated that in liver injury models, TREM2+ macrophages are recruited in injury liver and have been identified as essential for clearing damaged cells and mediating tissue repair (35).

This narrative review is based on the core pathogenesis of lipid metabolism disorder and immune response imbalance in MASH, taking TREM2 as the entry point. It summarizes the latest regulatory mechanisms of TREM2 in MASH, which may help develop new therapies for this disease.

## 2 Dysregulation of lipid metabolism and imbalance of immune response in MASH

**Figure 1:**
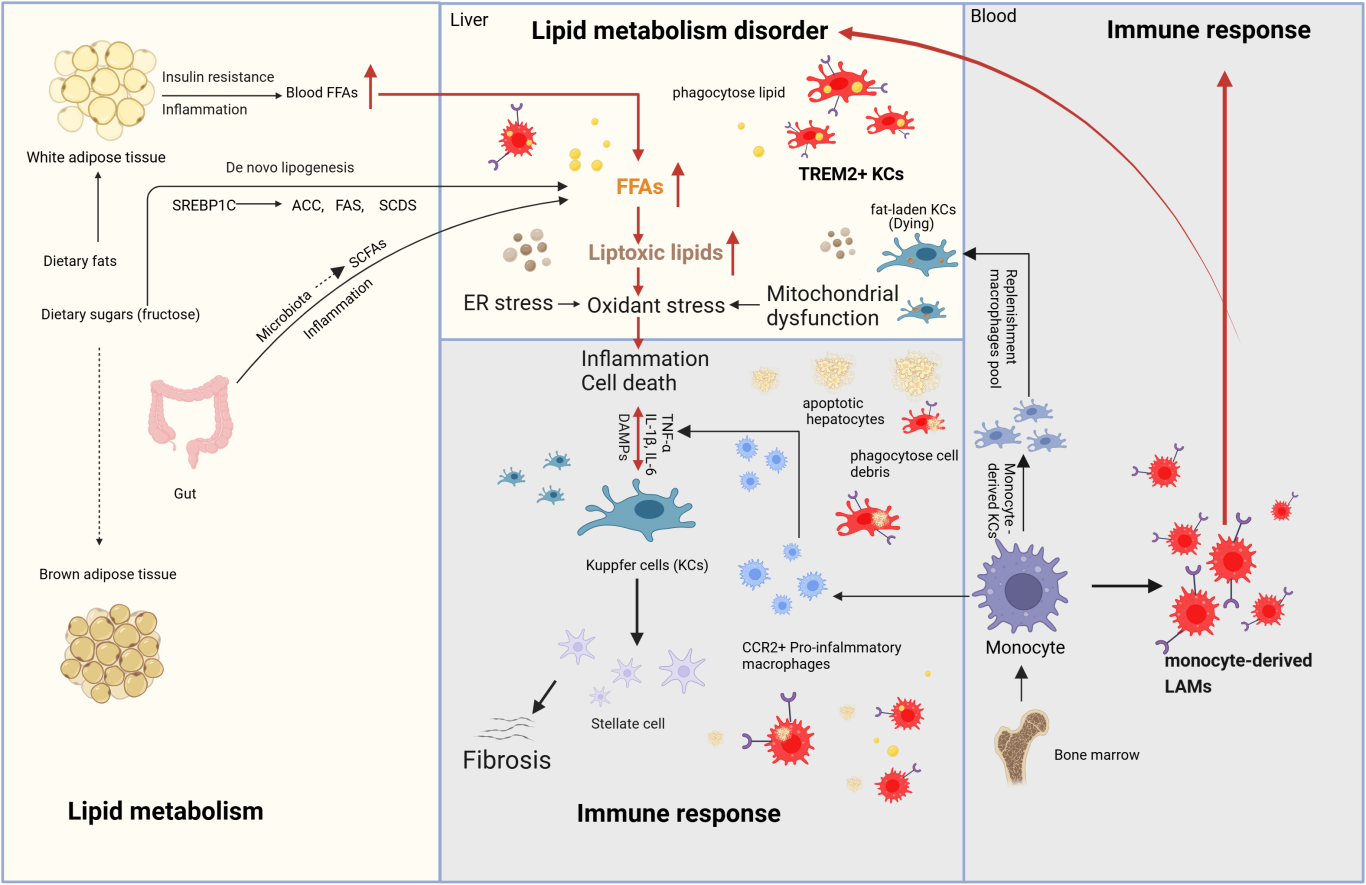
Mechanism of MASH (Metabolism and immune response). The main sources of fatty acids in the liver include fat mobilization from white adipose tissue, dietary intake, *de novo* lipogenesis from carbohydrates (especially fructose), and SCFAs generated by gut microbiota through the fermentation of undigested carbohydrates. During the progression of MASH, due to the dual effects of insulin resistance and inflammation, white adipose tissue generates fatty acids through lipolysis, which enter the bloodstream and subsequently reach the liver. *De novo* lipogenesis requires the participation of a series of enzymes, including ACC, FAS, and SCD1. These enzymes are regulated by SREBP-1c. The fatty acids generated through these pathways enter the liver, leading to continuous lipid accumulation and the formation of toxic lipids. The accumulation of toxic lipids causes ER stress, oxidative stress, and mitochondrial dysfunction in the liver, eventually resulting in hepatocyte death. DAMPs are molecules released during cell damage or death and serve as “signals” of cellular injury or stress. These molecules activate KCs, triggering liver immune responses and the release of inflammatory cytokines. Additionally, Kupffer cells can activate HSCs to release collagen fibers. Kupffer cells have another key characteristic that can phagocytose lipids and dead cells, ultimately leading to their exhaustion, while monocyte-derived macrophages from the bone marrow are recruited into the liver. On one hand, these monocyte-derived macrophages differentiate into CCR2+ pro-inflammatory macrophages and monocyte-derived KCs to replenish KC pool. On the other hand, with the focus of this review, they differentiate into TREM2+ macrophages, also known as LAMs, which regulate liver lipid metabolism, inhibit chronic liver inflammation and reverse the progression of MASH. Meanwhile, TREM2-expressing KCs are also present during MASH. MASH, Metabolic dysfunction-Associated Steatohepatitis; SCFAs, short-chain fatty acids; ACC, acetyl-CoA carboxylase; FAS, fatty acid synthase; SADS, stearoyl-CoA desaturase; SREBP-1c, sterol regulatory element-binding protein-1c; ER, endoplasmic reticulum; DAMPs, damage-Associated Molecular Patterns; TREM2, Triggering receptor expressed in myeloid cells 2; KCs, Kupffer cells; HSCs, hepatic stellate cells; LAMs, lipid-associated macrophages.

MASH is driven by a complex interplay of metabolic dysfunction, immune response (chronic inflammation), and fibrosis. Insulin resistance and dysregulated lipid metabolism lead to excessive hepatic lipid accumulation and lipotoxicity, triggering oxidative stress, mitochondrial dysfunction, and endoplasmic reticulum (ER) stress. These processes activate inflammatory pathways involving macrophages (KCs), and inflammatory cytokines (IL-1β, IL-6 and TNF-α), further exacerbating hepatocyte injury. Chronic inflammation stimulates HSCs activation, promoting fibrosis through transforming growth factor-β (TGF-β) and extracellular matrix deposition. Additionally, gut microbiota dysbiosis and genetic predisposition contribute to disease progression, ultimately increasing the risk of cirrhosis and HCC. The mechanisms mentioned above and their interactions are demonstrated in **Figure 1**.

### 2.1 lipid metabolism in MASH

The hepatocytes lipid metabolism dysregulation is the first step in MASLD development, which was be manifested in several key ways: 1) lipolysis in adipose tissue. Free fatty acids (FFAs) are primary delivered to the liver from the bloodstream following the lipolysis of triglyceride in adipose tissue. This process is regulated by the activation of insulin signal. When the post-receptor signal by insulin is impaired, it contributes to MASH by dysregulating lipolysis, resulting in excessive delivery of FFAs to the liver (36, 37). 2) *De novo* lipogenesis (DNL). In addition to being taken up by brown adipose tissue to exert thermogenic functions, dietary glucose and fructose serve as major substrates for *de novo* lipogenesis, providing a primary source of FFAs (38, 39), and this process involves a series of enzymes, including acetyl-CoA carboxylase (ACC), fatty acid synthase (FAS) and stearoyl-CoA desaturase (SCD1) and these enzymes are regulated by stearoyl-CoA response element binding proteion-1c (SREBP-1c) (40). When *de novo* lipogenesis increases beyond the liver’s metabolic capacity, it contributes to the occurrence of MASH. 3) Impaired fatty acid oxidation: In MASH, the liver’s ability to oxidize fatty acids is compromised, resulting in the accumulation of unoxidized fatty acids and the formation of fat droplets in liver cells (41).

### 2.2 immune response in MASH

Accumulating evidence suggests that immune response, especially the innate immune response of liver plays a pivotal role in the pathogenesis of MASH, driving chronic inflammation, hepatocellular injury, and fibrosis progression. The innate immune system serves as the first line of defense against various danger signals, including pathogen-associated molecular patterns (PAMPs) and damage-associated molecular patterns (DAMPs), both of which are critically involved in MASH pathophysiology. The liver harbors a diverse repertoire of innate immune cells, including the macrophage system (KCs and MoMFs), neutrophils, natural killer cells (NK cells), natural killer T cells (NKT Cells), HSCs, and innate lymphoid cells (ILCs). Here, we mainly focus on the alterations of the hepatic macrophage system in MASH.

#### 2.2.1 Kupffer cells in MASH

In human MASLD, macrophages are considered key players and an increased macrophages in periportal was an early histological hallmark (42). The liver-resident macrophages, KCs are the most abundant population of tissue resident macrophages in the human body, which are derived from the yolk sac, and replenished from circulating monocytes (43, 44). During MASH, overwhelming metabolic energy substrates lead to the accumulation of toxic lipid, production of reactive oxygen species (ROS), mitochondrial dysfunction and the induction of ER stress in the liver (45), and these cellular disturbances contribute to hepatocyte death and the subsequent release of DAMPs. KCs primarily act as sentinels, sensing metabolic stress and lipotoxic signals through pattern recognition receptors (PRRs) such as Toll-like receptors (TLRs), which recognize DAMPs and PAMPs derived from hepatocyte injury and gut-derived endotoxins. This activation leads to the downstream activation of Nuclear Factor kappa-light-chain-enhancer of activated B cells (NF-κB) and Nucleotide-binding oligomerization domain, leucine-rich repeat and pyrin domain-containing 3 (NLRP3) inflammasome, resulting in the release of pro-inflammatory cytokines (IL-1β, TNF-α) (**Figure 1**) (46). Additionally, during MASH, excessive free fatty acids, particularly palmitic acid and oxidized low-density lipoprotein (oxLDL), are taken up by KCs, leading to intracellular lipid accumulation and foam cell formation. This process impairs KCs phagocytic function. Excessive lipid uptake by KCs induces their apoptosis, then, KCs orchestrate monocyte infiltration through the secretion of C-C motif chemokine ligand 2 (CCL2), also known as monocyte chemoattractant protein-1 (MCP-1) and CCL5. Recruited monocytes differentiate into Monocyte-derived KCs, which further replenishment macrophages pool, amplify the inflammatory cascade and fibrogenic responses. KCs contribute to liver fibrosis by promoting HSCs activation via the secretion of TGF-β, platelet-derived growth factor (PDGF), and galectin-3 (47).

#### 2.2.2 Monocyte-derived macrophages in MASH

During the early stages of murine MASH, resident KCs partake in lipid storage, which ultimately renders them incapable of self-renewal, triggering their cell death. A series of studies recently demonstrated that in murine MASH, embryonic KCs are gradually lost and subsequently replaced by monocyte-derived KCs, one of the MoMFs, which are recruited to the liver from the bloodstream in response to chemotactic signals, particularly through the CCL2-CCR2 axis. MoMFs exhibit remarkable phenotypic plasticity, enabling them to adopt distinct functional phenotypes in response to various microenvironmental cues. In the early stages of MASH, stimulated by lipopolysaccharide (LPS), TNF-α and Interferon-gamma (IFN-γ), MoMFs are predominantly polarized toward the M1-like phenotype (C-C chemokine receptor type 2+ (CCR2) pro-inflammatory macrophages), which play a pro-inflammatory role by producing high levels of pro-inflammatory cytokine, contributing to the inflammatory microenvironment through generating ROS and nitric oxide (NO), resulting hepatocyte injury, and recruiting additional immune cells (**Figure 1**). As the disease progresses, they also play a role in the resolution of inflammation and tissue repair by polarizing towards the M2 phenotype induced by cytokines such as IL-4, IL-10, and IL-13. M2-like MoMFs can suppress excessive inflammation, enhance phagocytosis of apoptotic cells, and promotes fibrosis progression by supporting HSC activation. This shift is important for limiting excessive inflammation and promoting tissue regeneration.

#### 2.2.3 Lipid-associated macrophages in MASH

Recent single-cell RNA sequencing analyses have identified a population of TREM2-expressing macrophages, referred to as LAMs, in the white adipose tissue during obesity (25). During the progression of MASH, hepatic resident KCs participate in lipid storage, which ultimately compromises their ability to self-renew, leading to cell death. As a result, the resident KCs pool is replenished by monocyte-derived KCs recruited from the periphery. In murine steatohepatitis, infiltrating monocytes have at least 2 distinct fates: as monocyte-derived KCs, replenishing lost embryonic KCs, or as LAMs characterized by Cluster of Differentiation 9 (CD9) and TREM2 expression (**Figure 1**).

Spatial transcriptomics have revealed that TREM2+ macrophages are localized to sites of hepatocellular damage, inflammation, and fibrosis in the steatotic liver (48). Meanwhile, some studies found that this phenotype is not specific to recruited macrophages, as a subset of resident KCs can also adopt a LAM-like phenotype in the mouse and human liver. These TREM2+ KCs exhibit a hybrid M2-like phenotype, characterized by high expression of TREM2, Cluster of Differentiation 163 (CD163), and glycoprotein non-metastatic melanoma protein B (GPNMB), enhanced lipid metabolism, phagocytic activity and secretion of IL-10 and TGF-β. Several studies suggested that TREM2+ macrophages are considered specialized foam-like macrophages that facilitate cholesterol and lipid uptake through TREM2 signaling, alleviating hepatic lipotoxicity. Additionally, these TREM2+ macrophages (either monocyte-derived LAMs or TREM2+ KCs) maintain liver immune homeostasis by phagocytosing apoptotic or necrotic cells caused by lipotoxicity and oxidative stress and suppressing pro-inflammatory signaling pathways to decrease the production of pro-inflammatory cytokines. Thus TREM2+ macrophages play important roles in regulating lipid metabolism and immune homeostasis in the context of MASH in both humans and mice, making them a potential therapeutic target for MASH (48–51). Notably, different macrophage subsets may exert dual effects at various disease stages. In particular, the potential pro-fibrotic role of TREM2+LAMs in MASH has recently become a research focus.

## 3 TREM2 at the crossroads of metabolic and immune regulation

TREM2 has emerged as a pivotal regulator linking metabolic dysfunction to immune responses, particularly in chronic liver diseases such as MASH. Understanding its function and signaling pathways will provide a foundation for elucidating roles of TREM2.

### 3.1 The TREM family and its functions

Triggering receptors expressed on myeloid cells (TREM) family includes two members, TREM1 and TREM2, which their primary function is to recognize foreign antigens and toxic substances, thereby modulating the inflammatory response (52). TREM1 is expressed on KCs and HSCs in the liver and it play an important roles in promoting inflammation during acute inflammatory responses (53). In contrast, the TREM2 gene is predominantly expressed in myeloid cells (29). Under pathological conditions, TREM2 expression is generally upregulated. For instance, increased levels of TREM2 have been observed in patients with AD (54) and in mouse models of amyloid and tau pathology (55). Overexpression of TREM2 is thought to be associated with the recruitment of microglia to amyloid plaques (56). Additionally, upregulated expression of TREM2 has also been detected in aged mice and elderly humans. In the liver, TREM2 are expressed on KCs and cells including monocyte-derived macrophages and neutrophils, which infiltrate the liver in response to injury and TREM2 are overexpressed during MASH (20, 49). TREM2 has previously been shown to regulate lipid metabolism, inhibit chronic inflammation and promote cell survival which can protect liver from metabolic abnormalities, fibrosis, and tumorigenesis.

### 3.2 TREM2 ligands and signaling

TREM2 is a transmembrane receptor belonging to the immunoglobulin superfamily (57). Since TREM2 was initially identified in monocyte-derived dendritic cells *in vitro*, TREM2-dependent signaling has been widely demonstrated to be involved in immune-inflammatory responses across a variety of diseases (58). In recent years, the function of TREM2 and its corresponding signaling pathways have been extensively studied in the field of central nervous system (CNS) diseases, cancers and metabolic-associated diseases (29, 33, 50, 59, 60). TREM2 consist of an extracellular domain capable of recognizing diverse ligands. Studies on mouse macrophages showed that, due to the lack of any signal transduction or trafficking motifs in the cytoplasmic tail of TREM2, these proteins rely on DNAX-activation protein of 12kDa (DAP12) and DAP10 to transduce the signals (61, 62). When TREM2 interacts with its ligands, these co-receptors are phosphorylated, triggering downstream signaling pathways. Importantly, TREM2 interact with DAP12 or DAP10 to form TREM2-DAP12/DAP10 heterodimers, which are crucial for the transmission of TREM2 signaling and the execution of its function (63). The ligands of TREM2 encompass a wide array of molecules and materials, including DNA, lipoproteins, apolipoproteins (apolipoprotein E, APOE and apolipoprotein J, APOJ) and phospholipids (63, 64). Furthermore, TREM2 binds to ligands released as a consequence of tissue damage and cell death, such as, cell debris and LPS (64). Several studies on AD have revealed that TREM2 can directly interact with anionic and zwitterionic lipids, lipoproteins, and apolipoproteins (APOE, and APOJ) (65, 66). In AD mouse models, TREM2-deficient microglia show significantly reduced expression of APOE (67). Similarly, in high-fat diet (HFD) induced obesity mouse models, the expression of Lipoprotein Lipase (LPL) and APOE in adipose tissue macrophages is upregulated through a TREM2-dependent mechanism (25).

**Figure 2:**
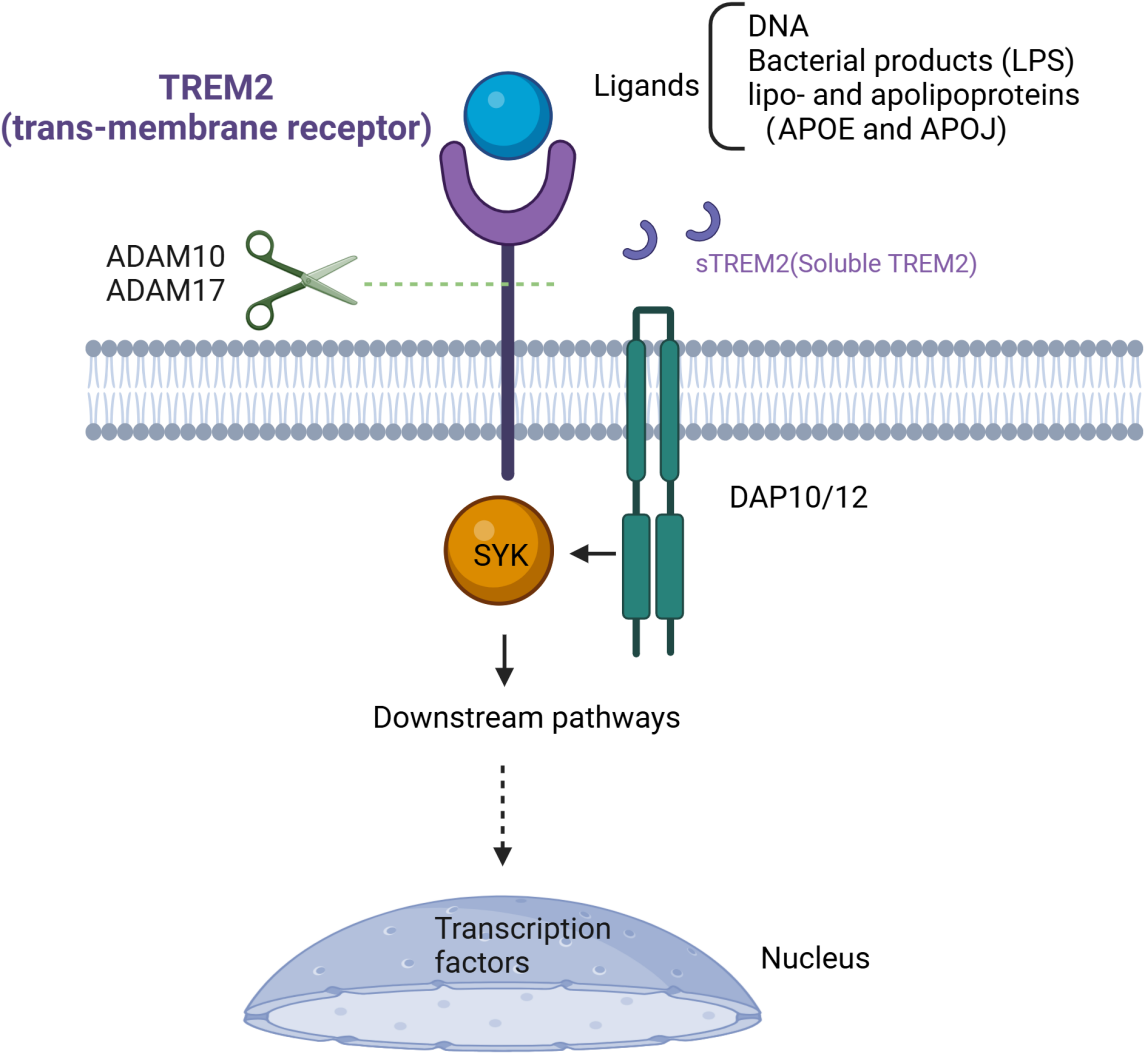
Targeting TREM2 signal pathway. TREM2, as a transmembrane receptor, requires the presence of DAP12 or DAP10 to exert its function after ligand binding. Its ligands include DNA, bacterial products (such as LPS), lipoproteins, and apolipoproteins (APOE, APOJ). Upon ligand binding, TREM2 recruits SYK protein, thereby activating a series of signaling pathways. Cleavage of TREM2 in the stalk region by ADAM10/17 creates sTREM2 and stops the TREM2 signaling cascade. TREM2, Triggering Receptor Expressed on Myeloid Cells 2; DAP12/10, DNAX-activation protein of 12kDa/10kDa; DNA, deoxyribonucleic acid; LPS, lipopolysaccharide; APOE, apolipoprotein E; APOJ, apolipoprotein J; ADAM10/17, A disintegrin and metalloproteinase10/17; sTREM2, soluble TREM2.

As mentioned above, a series of signal transduction events depend on the presence and the availability of DAP12. When DAP12 responds to the signal transmitted by TREM2, its immuno-tyrosine activation motif (ITAM) is phosphorylated by SRC tyrosine kinase (50, 63). As the ITAM region is phosphorylated, downstream tyrosine kinases such as Syk are activated, subsequently recruiting and activating various signaling molecules (**Figure 2**) (63). TREM2 signaling through DAP12 plays a crucial role in modulating lipid metabolism and immune response in MASH. Studies have found that TREM2 expression is abrogated by pro-inflammatory signaling of LPS, a TLR4 ligand or IFNγ (49, 68). Moreover, TREM2 shedding is a well-documented physiological event that occurs through a-secretases disintegrin and metalloproteinase domain-containing protein 17 (ADAM17) and ADAM10 cleaving human TREM2, resulting in production and release of soluble TREM2 (sTREM2) (**Figure 2**) (49, 69). The truncated transmembrane portion of TREM2 is then subject to further cleavage by γ-secretase, leading to its dissociation from DAP12 and subsequent signaling blockage (69).

In conclusion, the regulation of the TREM2 signaling pathway is an extremely complex process that dependent on the cellular state and tissue environment (**Figure 2**). While our understanding of TREM2 has advanced, further research is needed to focus on deciphering the interactions between TREM2 and its related ligands or downstream signaling pathways. Additionally, a deeper understanding of its involvement in a broader range of diseases will be crucial for advancing therapeutic strategies targeting TREM2-related pathways.

## 4 TREM2-mediated regulatory mechanisms in MASH pathogenesis

Based on current insights into the pathogenesis of MASH and the molecular signaling pathways of TREM2, the following section provide a detailed overview of the roles of TREM2+ macrophages in regulation of lipid metabolism and immune responses.

### 4.1 TREM2 is involved in lipid metabolism disorders in MASH

Studies have demonstrated that FFAs overload in hepatocytes causes lipotoxicity that damages hepatocytes (70, 71). Then, lipotoxic hepatocytes trigger the inflammatory response through the recruitment of immune cells. The lipid released from dead hepatocytes are subsequently taken up by activated KCs which will reduce lipid accumulation in the liver (72). In the early stage of steatohepatitis, toxic lipid metabolites accumulate in fat-laden KCs, this phenomenon promotes the production of inflammatory factors and weakens the self-renewal capacity of macrophages, leading to their exhaustion, which will replenished by MoMFs (monocyte-derived KCs) (**Figure 1**) (73).

**Figure 3:**
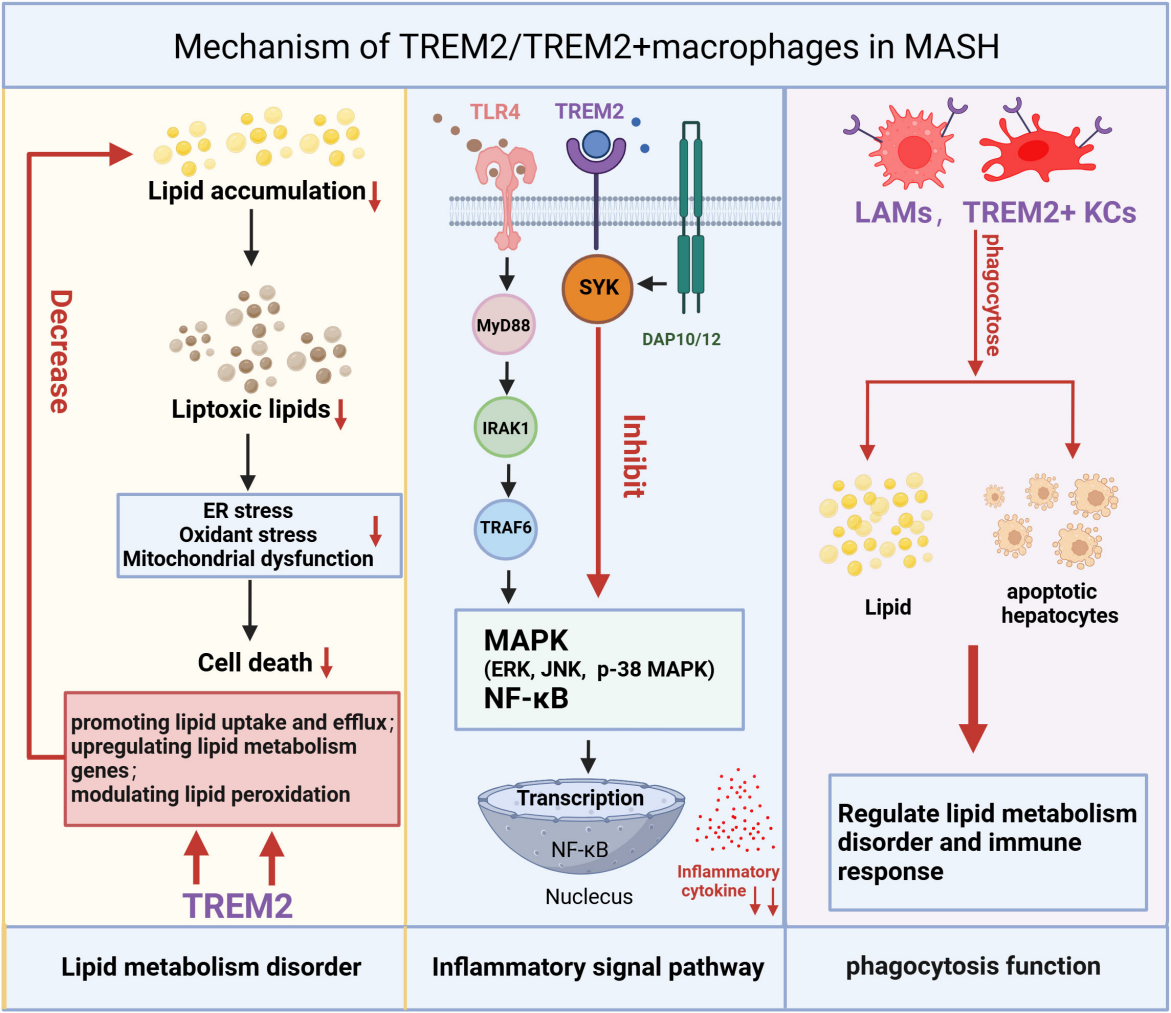
Mechanism of TREM2/TREM2+macrophages in MASH. The mechanism of TREM2/TREM2+ macrophages in MASH is reflected in three main aspects: regulation of lipid metabolism, inflammatory signaling pathways, and macrophage phagocytosis. TREM2/TREM2+ macrophages reduce lipid accumulation and toxic lipid-induced damage by enhancing lipid uptake, upregulating lipid metabolism-related genes, and modulating lipid peroxidation, thereby alleviating oxidative stress, ER stress, and mitochondrial dysfunction, ultimately reducing cell death. TREM2 can inhibit the inflammatory signaling pathway activated by the TLR4 (Upon ligand binding, TLR4 recruits MyD88 to its intracellular domain. MyD88 then recruits and phosphorylates members of the IRAK family, such as IRAK4 and IRAK1. Once IRAK1 is activated, it associates with TRAF6, a ubiquitin ligase that plays a central role in propagating the signal. TRAF6 subsequently triggers the activation of three major MAPK and NF-κB pathways). By inhibiting the MAPK and NF-κB pathways, TREM2/TREM2+macrophages can mitigate inflammatory responses. Additionally, TREM2+macrophages can phagocytose lipids and dead hepatocytes caused by oxidative stress, which is also a crucial step in regulating lipid metabolism disorders and immune responses. TREM2, Triggering Receptor Expressed on Myeloid Cells 2; ER stress, endoplasmic reticulum stress; TLR4, Toll-like receptor 4; MyD88, Myeloid differentiation primary response 88; IRAK, IL-1 receptor associated kinase; TRAF6, TNF receptor-associated factor 6; MAPK, Mitogen-Activated Protein Kinases.

**Figure 4:**
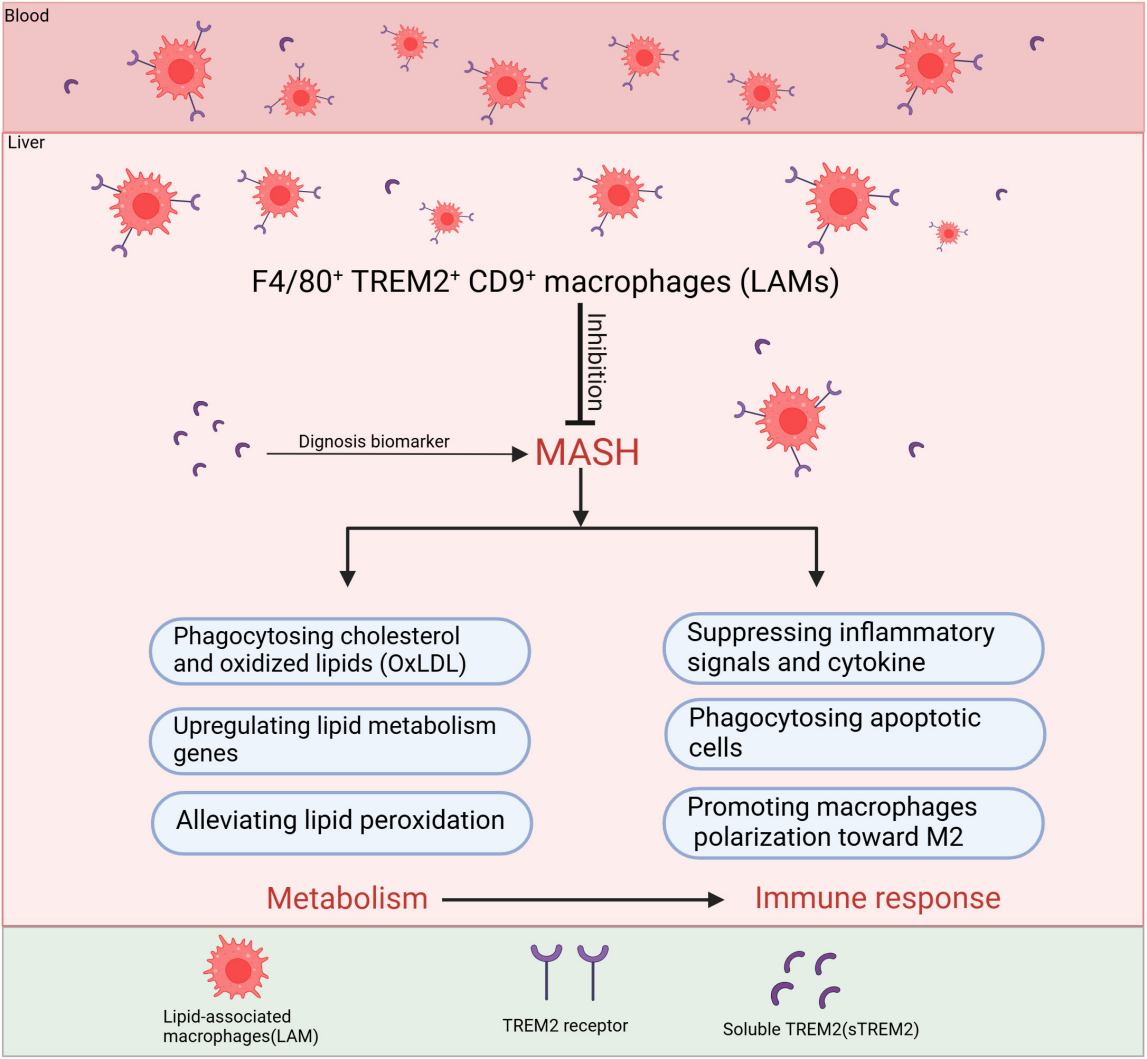
The main roles of TREM2+macrophages to inhibit the progression of MASH. F4/80^+^TREM2^+^CD9^+^ macrophages, also known as LAMs, migrate from the bloodstream into the liver and primarily inhibit MASH progression through two major mechanisms: regulating lipid metabolism and immune response. In terms of metabolic regulation, LAMs mainly phagocytose cholesterol and oxidized lipids, upregulate lipid metabolism genes and alleviate lipid peroxidation. Regarding immune regulation, LAMs inhibit the activation of inflammatory pathways, reduce the release of inflammatory cytokines, phagocytose dead cell debris and they also improve the disorder of metabolism to suppress inflammation. TREM2 can be cleaved by ADAM10/ADAM17, generating soluble TREM2 (sTREM2), which has been proven in clinical studies as a potential biomarker for the diagnosis and staging of MASH. TREM2, Triggering receptor expressed in myeloid cells 2; LAMs, lipid-associated macrophages; ADAM10/ADAM17, A disintegrin and metalloproteinase10/17; sTREM2, soluble TREM2.

TREM2+ macrophages, one of the cell populations differentiated from MoMFs, has been shown to regulate the uptake and metabolism of lipids, reducing fat deposition and helping to maintain hepatic lipid homeostasis (**Figures 3**, **4**) (74). A recent study explored the metabolic role of TREM2 by comparing TREM2-deficient (TREM2^-/-^) mice with wild-type (WT) littermates under normal chow or 8-week high-fat diet (HFD) conditions (74). While both groups on HFD exhibited expected metabolic perturbations, TREM2^-/-^ mice displayed exacerbated phenotypes of increased body and liver weight, systemic and hepatic lipid dysregulation and histopathological evidence of liver injury. The transcriptome analysis shows that gene sets related to fatty acid metabolic dysfunction, collagen fibril organization, and cytokine secretion were significantly enriched in TREM2^-/-^ livers (74). With the advancement of research, it has been discovered in recent years that TREM2 participates in the phagocytosis of lipid droplets, particularly in a subset of macrophages expressing both TREM2 and CD9 within white adipose tissue (LAMs) (75). TREM2, not only as a marker, but also as a driver of the LAM cell molecular program, TREM2-deficient macrophages are unable to phagocytose toxic lipids released from lipotoxic hepatocyte, which further lead to hepatocyte apoptosis, exacerbation of liver inflammation and fibrosis (25, 48). This lipid phagocytosis function may be due to TREM2 playing a critical role in regulating macrophage fusion and the formation of multinucleated giant cells (75). During the progression of MASH, the expression of TREM2+ macrophages increases, and they play a pivotal role in regulating lipid uptake and metabolism through TREM2-dependent signaling pathway (34, 48, 49). TREM2 interacts with lipid-associated molecules such as APOE to enhance macrophage phagocytosis of cholesterol and oxidized lipids. Additionally, it promotes cholesterol transport by activating the LXR-ABCA1/G1 axis, facilitating cholesterol efflux and preventing excessive cholesterol accumulation. This function of promoting lipid phagocytosis and cholesterol efflux has been further validated in microglia. In mice fed a cuprizone-containing diet, which induces demyelination, TREM2-deficient microglia have similar levels of phagocytosis of myelin debris compared to wild-type mice, but with insufficient cholesterol efflux, leading to the accumulation of cholesterol esters and oxidized cholesterol esters (76). In addition, TREM2 affects lipid peroxidation by changing metabolic pathways and ROS production in liver macrophages after liver injury (77). Furthermore, during the progression of MASH, the interplay between inflammation and metabolism is inextricable.

TREM2+ macrophages have been implicated in the pathogenesis of MASH through their dual roles in lipid metabolism and inflammation—specifically by upregulating lipid-processing genes, enhancing lipid uptake, and modulating peroxidation pathways (**Figures 3**, **4**). While targeting TREM2+ macrophages may represent a potential therapeutic strategy for MASH, it must be acknowledged that MAFLD/MASH itself can induce TREM2 expression. Thus, the upregulation of TREM2 might reflect a compensatory response to lipid overload. Notably, TREM2+ macrophages are known to regulate lipid metabolism in neurodegenerative disorders (e.g., AD). Avoiding systemic targeting of TREM2 may help prevent potential adverse effects on the central nervous system, and deeper mechanistic insights into how TREM2 directly regulates autophagy-related genes during MASH could advance precision medicine.

### 4.2 TREM2 is involved in immune imbalance in MASH

#### 4.2.1 Function of TREM2 in immune response

Liver immune response, especially chronic liver inflammation is another major characteristic in the progression of simple steatosis to MASH and HCC. In this dynamic immune response, liver macrophages including both resident KCs and monocyte-derived KCs play a dual role. On one hand, they can activate inflammatory signaling pathways and secrete pro-inflammatory cytokines, on the other hand, the state of hepatic macrophages also influences the progression of liver fibrosis (21, 23). Recent studies have shown that TREM2 suppresses inflammation by negatively regulating the synthesis of cytokines, such as TNF-α and IL-6, inhibiting the secretion of pro-inflammatory cytokines from KCs and helping HSCs to perform a protective role in overwhelming inflammation (50, 62, 63).

As mentioned above, LPS, an endotoxin and a component of the outer cell wall of Gram-negative bacteria can bind to TREM2 and activate downstream signaling pathways. LPS is a typical PAMP, used to induce inflammation, and it can regulate the expression of TREM2 indirectly (63). LPS-induced inflammation is usually mediated by Toll-like receptor 4 (TLR4), regulating the release of downstream inflammatory factors. This cascade of events, initiated by LPS/TLR4 signaling, impacts the expression of TREM2 and the activation of its function. Studies have indicated that during acute endotoxemia, LPS can decrease the expression of TREM2 (63). This is because a series of pro-inflammatory cytokines secreted upon activation of the LPS/TLRs pathway can suppress TREM2 expression. Additionally, the low expression of TREM2 is also attributed to its relatively short half-life. However, this situation is reversed in chronic inflammation, such as MASH, and CCL4 (Carbon Tetrachloride) induced liver injury (78). In chronic inflammation, KCs are polarized into anti-inflammatory M2-type KCs and protect the liver from chronic inflammation (79). In this context, the expression of TREM2 is upregulated and it binds to DAP12/DAP10 which negatively regulate inflammation to alleviate the liver injury. This upregulation of TREM2 may serve as part of the body’s compensatory mechanism to modulate the immune response, regulate inflammation, and promote tissue repair.

#### 4.2.2 Mechanism of TREM2 in immune response

Finally, the function of TREM2 suppressing signal pathways of inflammation and inhibiting the secretion of inflammatory cytokines is realized by antagonizing the downstream proteins associated with TLR4, such as p38 mitogen-activated protein kinase (p38-MAPK) and Extracellular signal-regulated kinase (ERK) (50, 62). TLRs are a family of pattern recognition receptors that play a pivotal role in the innate immune system by recognizing PAMPs and DAMPs (80–82). In humans, there are 10 different TLRs (TLR1 to TLR10), and the anti-inflammatory function of TREM2, consistent with its degradation mechanism is primarily mediated through TLR4 (68, 83). Upon ligand binding, TLR4 recruits Myeloid differentiation primary response 88 (MyD88) to its intracellular domain. MyD88 then recruits and phosphorylates members of the IL-1 receptor associated kinase (IRAK) family, such as IRAK4 and IRAK1. Once IRAK1 is activated, it associates with TNF receptor-associated factor 6 (TRAF6), a ubiquitin ligase that plays a central role in propagating the signal. TRAF6 subsequently triggers the activation of three major MAPK pathways: p38-MAPK, ERK, and c-Jun N-terminal kinase (JNK), which are critical for regulating inflammation and cell survival (84, 85). TREM2 reduces the activation of TLR4 pathway, such as p38-MAPK and ERK, which are critical for decreasing the production of pro-inflammatory cytokine (77). By suppressing TLR4 signaling, TREM2 helps prevent excessive inflammation in response to infection or injury, thereby protecting tissues from damage. TREM2-DAP12 is also involved in the regulation of the NF-κB (**Figure 3**) (86). Studies have demonstrated that the pro-inflammatory M1 phenotype to the anti-inflammatory M2 phenotype and inhibits osteoarthritis through the NF-κB/CXCL3 axis (87). Therefore, activation of the TREM2 signaling pathway can promote the polarization of macrophages towards the M2 phenotype, which are associated with their alleviation of the pro-inflammatory environment and the release of M2-related cytokines (IL-10, TGF-β) (87). M2 polarization contributes to inflammation resolution and tissue repair; however, it is important to note that this macrophage phenotypic shift may also promote fibrosis during MASH progression.

By influencing the aforementioned signaling pathways, TREM2 can inhibit the production of pro-inflammatory cytokine. TREM2-deficient mice show increased production of pro-inflammatory cytokines like TNF-α and IL-6, suggesting that TREM2 normally functions to dampen the inflammatory response. In the progression of MASH, the imbalance in lipid homeostasis, particularly the accumulation of toxic lipids, causing the activation of immune cells and inflammasomes, the secretion of pro-inflammatory cytokines, and increased oxidative stress exacerbate chronic inflammation. TREM2 helps in maintaining lipid homeostasis, preventing the excessive accumulation of lipids, that can decrease the progression of inflammation. Phagocytosis of apoptotic debris by TREM2+macrophages is also a mechanism to prevent secondary necrosis and release of endogenous pro-inflammatory danger signals. When hepatocytes undergo apoptosis or necrosis due to lipotoxicity, oxidative stress, or inflammation, they release specific signals (DAMPs) that attract TREM2+macrophages, including TREM2+ KCs and LAMs for phagocytosis. This process facilitates the clearance of necrotic and apoptotic hepatocytes in time, prevents the spread of inflammatory factors, and promotes the release of IL-10 and TGF-β, thereby reducing liver injury (**Figures 3**, **4**). Wang et al. found that TREM2+ macrophages can phagocytose apoptotic hepatocytes, thereby shut down immune activation and the progression of MASH (49). De Ponti et al. identified recruited LAMs and resident LAM-like KCs in multiple liver injury models and reveal that TREM2 expression on at least one of these is necessary to clear damaged cells, mediating tissue repair (35).

Although TREM2 plays a multifaceted role in modulating the immune response in MASH through the inhibition of pro-inflammatory signaling and pro-inflammatory cytokine, promotion of macrophage polarization toward M2 and phagocytosis of apoptotic hepatocytes (**Figure 3** and **4**), it is critical to acknowledge although TREM2+ macrophages are generally thought to possess the protective functions of M2-polarized macrophages, with the progression of inflammation, they may undergo a phenotypic shift toward M1-polarized macrophages. Therefore, further detailed and comprehensive studies of TREM2+ macrophage subsets are essential for addressing clinical challenges.

## 5 Potential diagnosis and therapeutic target

sTREM2, cleavage by ADAM10 or ADAM17 is detected in human cerebrospinal fluid (CSF) (88), and its levels are elevated in CSF of patients with various neurological conditions, such as AD, Parkinson’s disease (PD) (89–91). And matching CSF concentrations of sTREM2 with other measures of human brain function revealed that sTREM2 corresponds to a genetic AD risk status. Similar to TREM2+ macrophages, the expression level of sTREM2 is higher in MASH patients or mouse models compared to the non-MASH group. Recently, studies show that increased levels of sTREM2 appear earlier than other laboratory markers of MASH in both MASH patients and mouse models (48, 92). These markers could potentially serve as reliable noninvasive biomarker indicators for determining the stage and diagnosing the progression of MASH.

Given the role of TREM2 in regulating lipid metabolism and immune response, therapeutic strategies aimed at modulating TREM2 could provide novel approaches for managing MASH. 1). TREM2 agonists to regulate lipid metabolism and immune response. Compared with conventional broad-spectrum immunosuppressants such as CCR2/CCR5 antagonists and IL-1β blockers, targeting TREM2 offers superior safety and adaptability across different pathological stages of the disease. In the early stages of MASH, TREM2+macrophages improve lipid metabolic dysregulation by regulating lipid uptake. During hepatic inflammation, they contribute to the suppression of inflammatory signaling and promotes the transition to an anti-inflammatory macrophage phenotype. Therefore, although research on TREM2 is currently limited, modulation of TREM2 could provide a more effective and comprehensive strategy for addressing the disease than targeting CCR2/CCR5 or IL-1β. 2). Gene therapy to enhance TREM2 expression: Utilizing gene editing technology to increase TREM2 expression in liver macrophages, or to increase the proportion of TREM2+ macrophages in the liver can also play a role in anti-inflammation and reversal of MESH. 3). Targeting the gut-liver axis and TREM2: TREM2+ macrophages, which possess anti-inflammatory and tissue-repair properties, may be modulated by gut-derived signals. LPS may promote TREM2 expression, however, sustained exposure to high levels of LPS may drive hepatic macrophages toward a pro-inflammatory phenotype, thereby decreasing the expression of TREM2+macrophages and impairing their protective function. In addition, gut microbiota dysbiosis may drive TREM2+ macrophages toward a pro-inflammatory phenotype. Thus, balanced gut microbiota contributes to the maintenance of an anti-inflammatory immune environment, strategies that modulate gut microbiota or reduce gut permeability could indirectly influence the activation and function of TREM2 (93). This could be achieved using prebiotics, probiotics, or other therapies designed to balance the gut microbiome in MASH patients.

**Figure 5:**
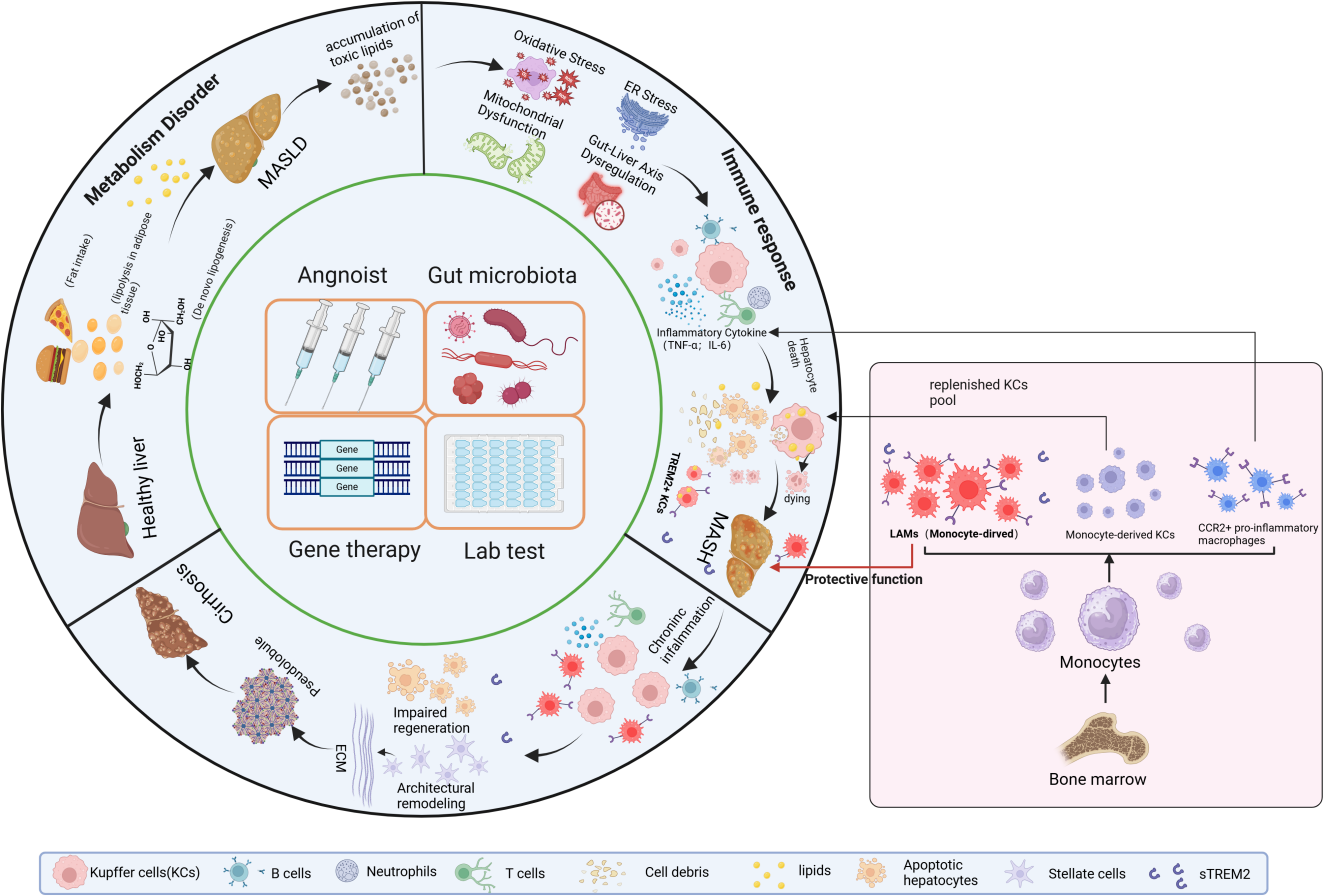
Overall Overview of MASH Progression and Clinical Strategies Targeting TREM2^+^ Macrophages4. TREM2 can delay disease progression at three stages: MASLD, MASH and cirrhosis. Clinical applications targeting TREM2 can be approached from four aspects: developing TREM2 agonists, modulating the gut microbiota to alter TREM2 expression in the liver, increasing the proportion of TREM2+ macrophages in the liver, editing TREM2-related genes. Additionally, sTREM2 can be used clinically for the diagnosis and staging of MASH. MASLD, Metabolic dysfunction-Associated Steatotic Liver Disease.

In conclusion, sTREM2 is an effective plasma biomarker to diagnosis MASH (**Figure 4**). And the potential clinical application strategies targeting TREM2 in MASH could provide novel approaches to modulate metabolic and immune homeostasis (**Figure 5**).

## 6 Discussion

Imbalance of lipid metabolism and immune homeostasis are central mechanisms in the development of Metabolic dysfunction-Associated Steatohepatitis (MASH) (6). The accumulation of fat leads to metabolic disturbances that trigger severe liver inflammation aggravating the progression of MASH. In recent years, the role of TREM2 in MASH has gained increasing attention as a critical mediator of both Metabolic and immune homeostasis (25, 48, 49). TREM2 expressing on the monocyte-derived LAMs or TREM2+ KCs plays a pivotal role in the regulation of liver lipid metabolism, immune response, and tissue repair in MASH. In this review, we first introduced the mechanisms of lipid metabolism and innate immune response in the development of MASH (**Figure 1**), and summarized the current research on TREM2 molecular signaling pathways (**Figure 2**). Based on this, we provided a detailed discussion on the role of TREM2+ macrophages in lipid metabolism and immune response regulation (**Figure 3** and **4**), mainly focused on the function of lipid phagocytosis and suppressing chronic inflammation during MASH (25, 63, 87).

Despite significant advances in understanding the mechanisms of TREM2 in MASH, further studies are required to fully elucidate its precise roles and molecular mechanisms. As we all know, gut–liver axis plays a pivotal role in the pathogenesis of MASH, primarily through translocation of microbial-derived products such as LPS, bile acids, and short-chain fatty acids (SCFAs). Gut-derived endotoxins can activate liver macrophages and promote inflammation, however, chronic low-dose LPS stimulation in combination with the products of gut probiotics may contribute to the maintenance of an anti-inflammatory microenvironment. Thus, TREM2, through its interaction with gut microbial metabolites, may modulate macrophage responses to gut-derived signals, potentially influencing the liver metabolism and immune response in MASH. The liver–brain axis refers to the bidirectional communication network between the liver and the brain, involving complex interactions between the liver, gut, and CNS. The functions of this axis include the regulation of energy metabolism, immune responses, and neurological functions. In recent years, an increasing body of research has highlighted the critical role of the liver–brain axis in metabolic diseases (94). TREM2 has been shown to play an important role in the progression of CNS diseases. Therefore, it is essential to explore the regulatory role of TREM2 in the gut-liver axis and liver–brain axis in the future, offering new therapeutic strategies for MASH. However, it is worth noting that systemic modulation of TREM2 may induce CNS or other system-related side effects. Therefore, we highlight the urgent need to develop precise therapeutic strategies targeting TREM2. 1): The integration of nanotechnology with TREM2 agonists facilitates their targeted delivery to liver tissue, improving the specificity and precision of TREM2 modulation. 2): Using single-cell sequencing technology identifies tissue-specific TREM2^+^ macrophage subsets. 3): It is needed to identify the key molecular mechanisms and avoid continuous modulation of TREM2 throughout the progression of MASH.

Surprisingly, a recent study reported a new pro-fibrotic role of TREM2, which contrasts with its well-characterized anti-inflammatory and tissue-protective functions during earlier stages of MASH progression (95, 96). While TREM2+ macrophages are generally considered reparative, their sustained activation in an inflammatory and lipid-rich microenvironment may adopt a pro-fibrotic phenotype. These cells can secrete profibrotic mediators such as TGF-β and PDGF which in turn activate HSCs and promote extracellular matrix deposition. So, in the early stages of MASH, the upregulation of TREM2 may represent a hepatic compensatory response aimed at clearing excessive lipids and attenuating liver inflammation. However, in chronic or advanced stages, the upregulation of TREM2 may shift toward a pathological role, contributing to hepatic fibrosis and exacerbating disease severity. Therefore, achieving stage-specific modulation of TREM2 signaling are essential. Using the biomarker sTREM2 and other clinical tools to accurately stage patients and the stage-adapted therapeutic strategy combined with appropriate antifibrotic agents may optimize the clinical management of MASH. Furthermore, a more comprehensive and in-depth understanding of the role and the mechanisms that TREM2+ macrophages contribute to fibrosis remain to be fully elucidated to ensure the safe and effective translation of TREM2-targeted therapies into clinical practice.

## 7 Conclusion

TREM2+ macrophages represent a promising therapeutic target in MASH due to their dual role in regulating lipid metabolism and immune response (**Figure 5**). Further investigations into their precise functions and mechanism in the context of metabolic liver disease will be essential to advance our understanding and treatment of MASH.

## Glossary

MASH: Metabolic dysfunction-Associated Steatohepatitis
MASLD: Metabolic dysfunction-Associated Steatotic Liver Disease
HCC: Hepatocellular carcinoma
T2DM: Type 2 diabetes mellitus
CVD: Cardiovascular disease
CKD: Chronic kidney disease
HSCs: Hepatic stellate cells
IL-1β: Interleukin-1β
IL-6: Interleukin-6
TNF-α: Tumor Necrosis Factor-α
KCs: Kupffer cells
MoMFs: Monocyte-derived macrophages
TREM2: Triggering Receptor Expressed on Myeloid Cells 2
AD: Alzheimer’s disease
ER: Endoplasmic reticulum
TGF-β: Transforming growth factor-β
FFAs: Free fatty acids
DNL: *De novo* lipogenesis
ACC: Acetyl-CoA carboxylase
FAS: Fatty acid synthase
SCD1: Stearoyl-CoA desaturase
SREBP-1c: Stearoyl-CoA response element binding proteion-1c
PAMPs: Pathogen-associated molecular patterns
DAMPs: Damage-associated molecular patterns
NK cells: Natural killer cells
NKT Cells: Natural killer T cells
ILCs: Innate lymphoid cells
ROS: Reactive oxygen species
PRRs: Pattern recognition receptors
TLRs: Toll-like receptors
LPS: Lipopolysaccharides
TLRs: Toll-like receptors
NF-κB: Nuclear Factor kappa-light-chain-enhancer of activated B cells
NLRP3: Nucleotide-binding oligomerization domain, leucine-rich repeat and pyrin domain-containing 3
CCL2: C-C motif chemokine ligand 2
PDGF: Platelet-derived growth factor
IFN-γ: Interferon-gamma
CCR2: C-C chemokine receptor type 2
NO: Nitric oxide
LAMs: Lipid-associated macrophages
CD9: Cluster of Differentiation 9
CNS: Central nervous system
DAP12: DNAX-activation protein of 12kDa
DAP10: DNAX-activation protein of 10kDa
APOE: Apolipoproteins E
APOJ: Apolipoprotein J
HFD: High-fat diet
LPL: Lipoprotein Lipase
ITAM: Immuno-tyrosine activation motif
ADAM17: A-secretases disintegrin and metalloproteinase domain-containing protein 17, sTREM2, Soluble TREM2
p38-MAPK: p38 mitogen-activated protein kinase
ERK: Extracellular signal-regulated kinase
MyD88: Myeloid differentiation primary response 88
IRAK: IL-1 receptor associated kinase
TRAF6: TNF receptor-associated factor 6
JNK: c-Jun N-terminal kinase
CSF: Cerebrospinal fluid
PD: Parkinson’s disease.
